# scDual-Seq: mapping the gene regulatory program of *Salmonella* infection by host and pathogen single-cell RNA-sequencing

**DOI:** 10.1186/s13059-017-1340-x

**Published:** 2017-10-27

**Authors:** Gal Avital, Roi Avraham, Amy Fan, Tamar Hashimshony, Deborah T. Hung, Itai Yanai

**Affiliations:** 1 Institute for Computational Medicine and Department of Biochemistry and Molecular Pharmacology, New York University School of Medicine, New York, NY 10016 USA; 2 Department of Biology, Technion – Israel Institute of Technology, Haifa, 32000 Israel; 3 Broad Institute of Harvard and MIT, Cambridge, MA 02142 USA; 4 Department of Molecular Biology and Center for Computational and Integrative Biology, Massachusetts General Hospital, Boston, MA 02114 USA; 5 Department of Genetics, Harvard Medical School, Boston, MA 02115 USA; 6 Present address: Department of Biological Regulation, Weizmann Institute of Science, Rehovot, Israel

## Abstract

The interaction between a pathogen and a host is a highly dynamic process in which both agents activate complex programs. Here, we introduce a single-cell RNA-sequencing method, scDual-Seq, that simultaneously captures both host and pathogen transcriptomes. We use it to study the process of infection of individual mouse macrophages with the intracellular pathogen *Salmonella typhimurium*. Among the infected macrophages, we find three subpopulations and we show evidence for a linear progression through these subpopulations, supporting a model in which these three states correspond to consecutive stages of infection.

**Electronic supplementary material:**

The online version of this article (doi:10.1186/s13059-017-1340-x) contains supplementary material, which is available to authorized users.

## Background

**Fig. 1:**
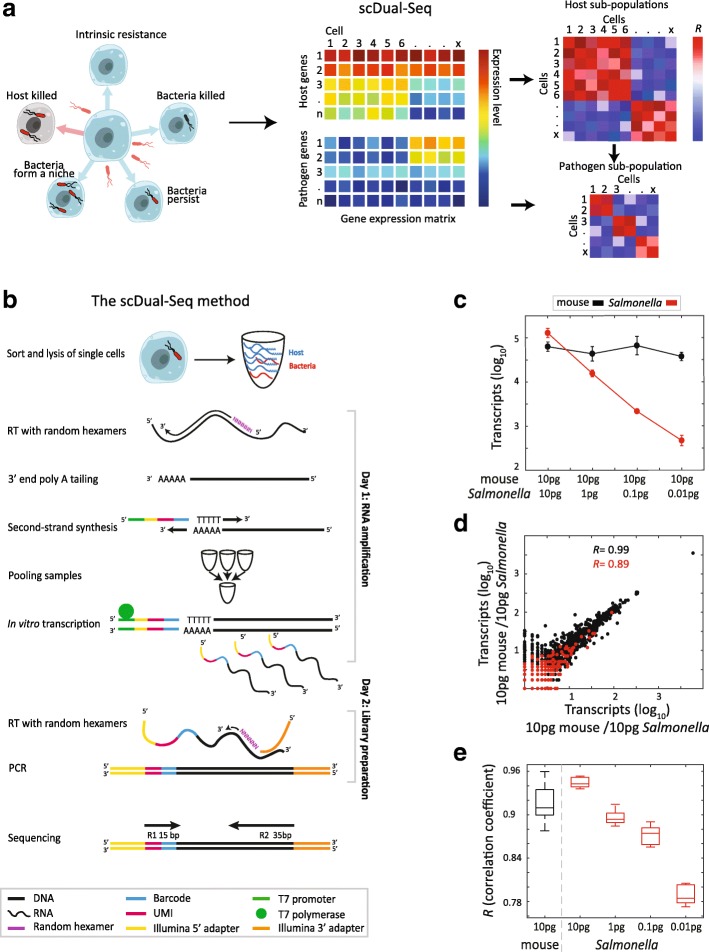
A single-cell RNA-sequencing approach to studying host–pathogen interaction. **a** Heterogeneity of outcomes of intracellular infection is due to both *Salmonella* and macrophage states. scDual-Seq simultaneously produces the transcriptome of both the host and the pathogen and allows the identification of cellular subpopulations during infection. **b** *Schematic* of the scDual-Seq method. Reverse transcription is primed using random hexamers, followed by RNase treatment and 3’ polyA tailing. The second strand is synthesized using the CEL-Seq2 barcoded primers (see “Methods”). The samples are pooled together before the complementary DNA (cDNA) undergoes linear amplification by in vitro transcription. The amplified RNA is then reverse transcribed using a random primer with an overhang of the sequence complementary to the Illumina 3’ adaptor. cDNA with both Illumina adaptors are selected by polymerase chain reaction and the DNA library is sequenced using paired-end Illumina sequencing. **c** Mean number of unique transcripts identified across five technical replicates, for mouse (*black*) and *Salmonella* (*red*). *Circles* and *error bars* represent the mean and standard deviation. **d** *Plot* between the expression of the two technical replicates of 10 pg mouse RNA and 10 pg *Salmonella* RNA. **e** *Boxplots* indicating the correlation coefficients across replicates with the sum expression of all 20 samples for mouse and for five replicates in each dilution for *Salmonella*. Mouse indicated in *black*, *Salmonella* dilutions indicated in *red*

The rise of antibiotic-resistant bacterial pathogens constitutes one of the most serious threats to human health. Many of these resistant pathogens, such as *Mycobacterium tuberculosis*, *Salmonella enterica*, and *Neisseria gonorrhea*, spend a significant portion of their life-cycle surviving and replicating within host cells, typically macrophages [1]. The interaction between a pathogen and a host is a highly dynamic process in which both orchestrate intricate gene regulatory pathways. For example, the pathogen *S. enterica* stimulates the inflammatory response of the body, invades macrophages, and alters their gene expression in order to optimize its survival conditions [2]. During infection, however, multiple outcomes are observed when a bacterium encounters a host cell, including bacterial clearance, bacterial survival and persistence, or host cell death (Fig. 1a). These different phenotypic outcomes thus suggest heterogeneous cellular behavior [3, 4], which makes a single-cell approach crucial for the dissection of the factors contributing to the different infection outcomes.

While simultaneous transcriptome analysis of host and bacteria by RNA-sequencing (RNA-seq) can provide a comprehensive view of cellular states, previous efforts using bulk measurements have been limited to averaging over thousands of host and pathogen cells [5], thereby losing the ability to capture the heterogeneity of the individual encounters. Another major limitation in single-cell RNA-seq is the dependence on oligo-dT priming, which has restricted the examination to only individual host cells since this approach does not provide information on the state of the individual infecting pathogen [5–8]. Although poly-A independent methods for single cells are available [9, 10], their efficacy for detecting intracellular bacterial transcriptomes is untested.

Here, we present scDual-Seq, a method to simultaneously analyze both host and pathogen cells using single-cell RNA-seq. This method is highly multiplexed, uses UMI to allow single transcripts quantification, and takes advantage of in vitro transcription (IVT) for amplification. We show that by using scDual-Seq, we are able to identify and quantify the expression levels of both bacterial and host transcripts in individual infected mammalian cells.

## Results and discussion

We developed a single-cell dual capture and sequencing method ─ scDual-Seq ─ for single-cell RNA-seq to analyze simultaneously both host and bacterial cells. To capture both bacterial and host transcripts, scDual-Seq primes the reverse transcription (RT) reaction using random hexamer DNA oligos (see “Methods,” Fig. 1b, and Additional file 1). After adding a polyA tail to the complementary DNA (cDNA), scDual-Seq proceeds as an extensive modification of the CEL-Seq2 method for single-cell RNA-seq [11, 12], which includes barcoding for multiplexing, IVT for amplification, and paired-end Illumina sequencing.

To assay the ability of scDual-Seq to detect transcripts of both eukaryotic and bacterial origin, we processed RNA extracted from bulk samples of mouse embryonic stem cells (mESC) and from the Gram-negative, intracellular pathogen *S. typhimurium* (*Salmonella*). On average, we detected 63,000 unique mouse transcripts and 120,000 unique *Salmonella* transcripts when starting with 10 pg RNA, the estimated amount of RNA present in a mammalian cell, respectively (Fig. 1c). This is considerable given that one mESC cell is thought to consist of 500,000 transcripts [13]. To study the sensitivity of scDual-Seq with reduced RNA input amounts, we processed samples with 10 pg of mESC RNA and 1 pg, 0.1 pg, and 0.01 pg *Salmonella* RNA, respectively. We detected roughly the same number of mouse transcripts and a decrease of one order of magnitude in *Salmonella* transcripts across the dilutions, as expected from the linearity of detection in scDual-Seq (Fig. 1c). Due to the random priming during RT, we detected messenger RNAs (mRNAs) and non-coding RNA in our samples (Additional file 2: Figure S1a). While most of the *Salmonella* transcripts correspond to non-coding RNA, in mouse this is not the case; which may be attributed to a difference in the structure of the prokaryotic and eukaryotic ribosomal RNAs. We further detected high correlations between technical replicates; *R* = 0.99, on average, for 10 pg mouse samples and *R* = 0.89 for the 10 pg *Salmonella* samples (Fig. 1d shows one pair of technical replicates). The reproducibility, however, is reduced with lower input amounts: for 0.01 pg *Salmonella* RNA, the average correlation is 0.79 (Fig. 1e). Based on these studies, we concluded that scDual-Seq accurately measures RNA levels in samples containing as little as 0.01 pg RNA for both polyA+ and polyA- RNA. On average, we detected 470 *Salmonella* transcripts in 0.01 pg of RNA, which is the expected amount of RNA in a single bacterial cell [14]. Since this amount of RNA has been estimated to correspond to 10,000 transcripts, scDual-Seq has an estimated sensitivity of approximately 4.7%.

To test for the sensitivity of scDual-Seq in measuring the transcriptomes of live *Salmonella*, we identified genes differentially expressed between an overnight culture of *Salmonella* grown in bulk and intracellular *Salmonella* within macrophages in exposed single cells, and 10 and 100 cell populations. We detected a similar set of differentially expressed genes in all three comparisons, indicating that sensitivity is not severely compromised at the single-cell level (*P* < 0.001; hypergeometric distribution; Additional file 2: Figure S1b). Second, we found good correspondence of *Salmonella* transcriptomes between the single-cell data and population-level data, as well as between the 10-cell and 100-cell population data (Additional file 2: Figure S1f), demonstrating the accuracy of the single-cell measurements of bacterial transcripts. Comparing the sensitivity of scDual-Seq directly with that of CEL-Seq2, we found that CEL-Seq2 has higher sensitivity with more detected mouse genes than scDual-Seq (Additional file 2: Figure S1c). However, examining at the number of detected *Salmonella* genes (non-polyA), scDual-Seq performed better than CEL-Seq2. scDual-Seq shows the same dependency of noise on expression level that was observed in CEL-Seq [11] (Additional file 2: Figure S1d, e).

**Fig. 2:**
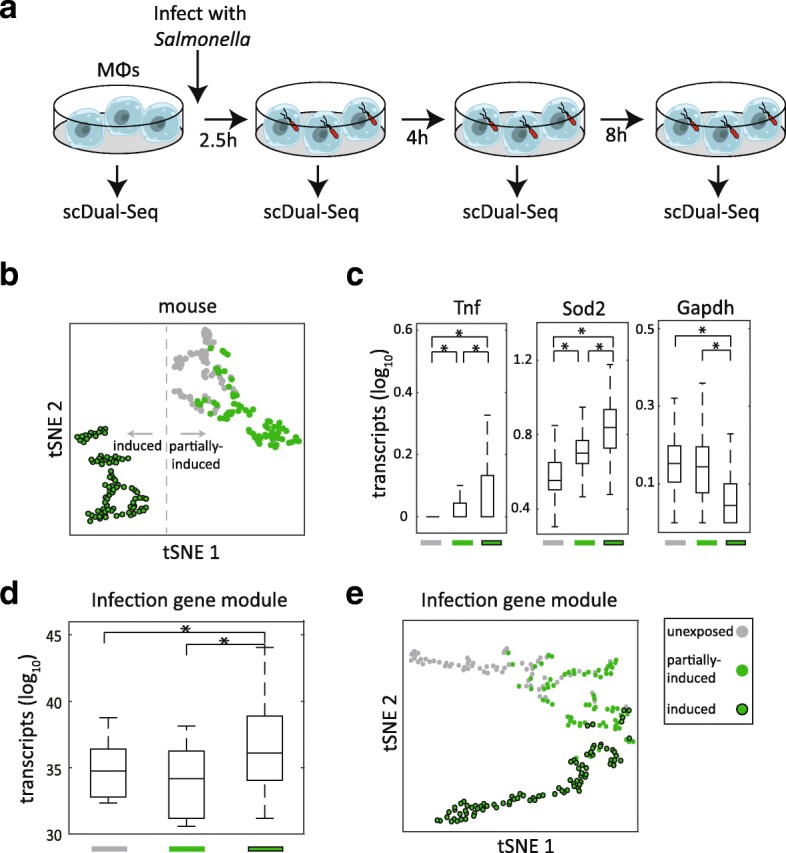
Identifying host subpopulations in mouse macrophages exposed to *Salmonella*. **a** Bone marrow-derived macrophages, exposed or unexposed to *Salmonella*, were sorted into a 96-well plate and processed using scDual-Seq at the four indicated time-points. **b** tSNE *plot* of single cells (perplexity = 10) computed based on correlation matrix between single cells using 457 mouse genes with high expression variation (mean/median > 1) and maximum expression higher than 10 tpm. The color indicates exposed (*green*), unexposed (*gray*), and induced (*black circle*). DBscan was used to cluster the cells into two groups. **c** *Boxplot* of expression levels (log_10_ tpm) of the indicated mouse genes across the non-infected, partial-induced, and induced single cells (*P* < 0.02, Wilcoxon rank sum test). **d** *Boxplots* of the sum expression of the previously reported infection gene module [3] across the exposed and unexposed individual cells in our scDual-Seq data (*P* < 0.0001, Wilcoxon rank sum test). **e** tSNE *plot* of single cells (perplexity = 10) computed based on the normalized expression of the previously reported infection gene module [3]. Cells were colored according to their annotation in (**a**)

Previous work has identified that infection is accompanied by significant and dramatic gene expression changes in either the host or the pathogen [5, 15, 16]. To simultaneously query both host and pathogen transcriptomes, we isolated mouse bone marrow-derived macrophages and analyzed: (1) infected cells that were isolated by fluorescence-activated cell sorting (FACS) after having been exposed to GFP-expressing *Salmonella* at a 50:1 MOI, at three time-points (2.5, 4, and 8 h after infection); and (2) cells that were not exposed to the pathogen (unexposed), as control (Fig. 2a, Additional file 2: Figure S2a–c, see “Methods”). For each time point, 96 cells were processed using scDual-Seq.

By performing an unbiased comparison of the macrophage single-cell transcriptomes across the time-points, we identified two responses that, for the most part, distinguished the infected and unexposed cells (Fig. 2b, Additional file 2: Figure S2d). Among the infected cells, one type of response characterizes the majority of infected cells that we term an induced response (87 cells). A second type of response characterizes the 69 infected cells whose transcriptome resembles that of unexposed cells, which we term a “partially induced” response because their global transcriptional patterns are more similar to the unexposed than other infected cells. Genes known to be part of the immune response – *Tnf* and *Sod2* – are differentially expressed between the unexposed and partially induced cells relative to the induced cells. As a control, we verified that *Gapdh* expression levels were not also higher in the induced cells (Fig. 2c). Moreover, genes previously detected to be differentially expressed between exposed and unexposed macrophages [3] show significant expression differences across these clusters (Fig. 2d). While the partially induced cells do not induce a full immune response in contrast to the induced cells, they may nonetheless be considered as having a “partial response” because as a population they have higher expression (though not significant) of some major immune response genes when compared to the unexposed cells, e.g. *Tnf* (Fig. 2c). Furthermore, we visualized the relations among the identified groups based upon the aforementioned immune response genes [3]. Strikingly, we found an arc-shaped distribution of cells whereby the partially induced cells are flanked by the unexposed and induced cells (Fig. 2e, Additional file 2: Figure S2e).

**Fig. 3:**
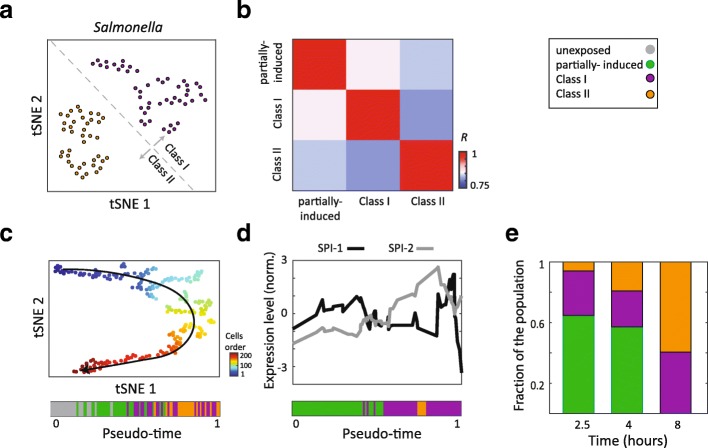
A time-course of *Salmonella* infection. **a** tSNE *plot* of induced single cells (perplexity = 10) computed based upon 32 *Salmonella* regulons. Color indicates Class I (*purple*) and Class II (*orange*). DBscan was used to cluster the cells into two groups. **b** The *heatmap* indicates Pearson’s correlation coefficients between the average *Salmonella* regulons expression of the partially induced, Class I, and Class II subpopulations. **c** tSNE *plot* (positioned as in Fig. 2e) colored by the pseudo-time order of the cells. The bar below indicates unexposed (*gray*), partially induced (*green*), Class I (*purple*), and Class II (*orange*) cells, ordered by pseudo-time. **d** *Plot* of normalized expression level of SPI-1 and SPI-2 regulons in single cells ordered according to pseudo-time and smoothed (see “Methods”). The bar below indicates partially induced (*green*), Class I (*purple*), and Class II (*orange*) cells, ordered by pseudo-time. **e** *Bar chart* indicating for each time-point (2.5, 4, and 8 h after infection), the fraction of the three identified subpopulations (partially induced, Class I, and Class II)

We next sought to study *Salmonella* expression in the induced cells. Since *Salmonella* expression is relatively sparse in terms of the number of detected transcripts, we pooled the expression of genes belonging to the same regulon [17] into a collective expression profile (see “Methods”). While these regulons are partially defined based upon *E. coli* orthology [17] – in addition to *Salmonella*-based experiments – it has been previously shown that co-expression among genes is generally evolutionarily conserved [18, 19]. Examining the expression of the *Salmonella* regulons within the induced macrophages of all three time-points, we identified two classes of intracellular *Salmonella* with distinct transcriptional signatures which we denote Class I (47 single cells) and Class II (40 single cells) (Fig. 3a). Examining mouse gene expression and *Salmonella* regulon expression specific to each class revealed high-virulence functionality in Class I *Salmonella* (Additional file 2: Figure S3a, b). We further confirmed this separation into two *Salmonella* classes as well as the cluster of partially induced cells in an independent experiment involving a single time-point (Additional file 2: Figure S4a–c).

**Fig. 4:**
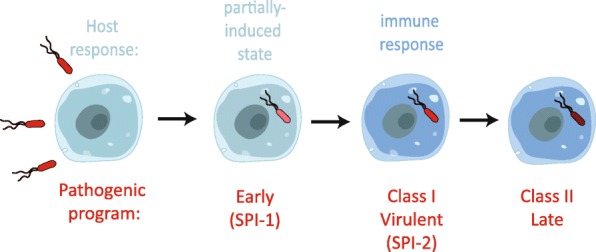
A model of consecutive infection stages. A model for the gene regulatory program of *Salmonella* infection. Host and pathogen transcriptomic processes are indicated in *blue* and *red*, respectively

To gain further insight into the molecular composition of the subpopulations, we compared gene expression across the partially induced, Class I, and Class II cells, according to their *Salmonella* regulon expression profiles. Surprisingly, Class I subpopulation is more correlated to that of the *Salmonella* regulon expression in partially induced cells than to that of Class II cells, despite the fact that both Class I and II *Salmonella* induce the same response from the host (Fig. 3b, *P* < 0.0001, Wilcoxon rank sum test, see “Methods”). There are two main possible models to explain the simultaneous observation of these three subpopulations (the partially induced macrophages infected with Class-I-like *Salmonella*, the fully induced macrophages infected with Class I *Salmonella*, and the fully induced macrophages infected with Class II *Salmonella*). In the first, the three different subpopulations could represent different stages through which infection progresses linearly (Fig. 4). While the host cells were exposed to *Salmonella* simultaneously, the rate of infection progression could be non-uniform, resulting in the co-existence of all three subpopulations. Alternatively, the co-existence of the three subpopulation classes may indicate that infection proceeds via different parallel, individual programs wherein a commitment to a particular state of infection is made by the encounter between an individual bacterium and host cell.

To distinguish between these two models, we used pseudo-time analysis based upon the tSNE plot in Fig. 2e. Pseudo-time was inferred based upon the Euclidean distance among the cells (in tSNE space, Fig. 3c, see “Methods”). Interestingly, we observed that the ordered cells recapitulated the clustering observed in the previous analysis. Cells of the same group generally clustered: first the unexposed cells, next partially induced cells followed by Class I cells and finally Class II cells. To test whether ordering by pseudo-time reflected known biological processes, we queried for the SPI-1 and SPI-2 (type-III secretion systems) *Salmonella* regulons which are known to be inversely expressed during infection [20–22]. Interestingly, we found that while the mean expression of both regulons is similar across the subpopulations (Additional file 2: Figure S3B), examining their expression level at the pseudo-time single-cell level ordered cells, SPI-1 is more highly expressed in the partially induced cells, early in the infection, and SPI-2 is more highly expressed in Class I later in infection (Fig. 3d, see “Methods”). This order of SPI-1 and SPI-2 expression matches that from previous reports [20–22].

To further query whether the infection progresses linearly, we followed the distribution of these subpopulations over the time course of infection. If the linear model is correct with the partially induced cells representing the earliest state in infection, we would expect that the fraction of the cells that are partially induced to decrease with time. Indeed, calculating the fraction of each subpopulation at each time point, we found the largest fraction of partially induced cells at 2.5 h after infection, with a smaller fraction at 4 h, and no partially induced cells at all at 8 h (Fig. 3e). This result, as well as the pattern of correlations among the three subpopulations (Fig. 3b) and the order of cells according to the infection pseudo-time (Fig. 3c), is consistent with infected macrophages following a linear progression from the partially induced state to the fully induced state process (Fig. 4). Further, since the Class I *Salmonella* transcriptomes in the fully induced macrophages are transcriptionally more akin to those of the *Salmonella* in partially induced cells, this could suggest a progression of the partially induced cells to the fully induced cells containing Class I *Salmonella*. At this stage, the host response is induced in a large-scale upregulation of immune functions. Finally, infection could then proceed to macrophages maintaining an induced state with *Salmonella* in a Class II state. Since the linear and parallel models need not necessarily be mutually exclusive, a combination of these two—a temporal linear process in some cells such as the partially induced macrophages and parallel commitments to different strategies in others—may be possible.

## Conclusions

scDual-Seq represents a novel method to query host–pathogen interactions. We expect that among its many possible uses, it will be invaluable to study the adaptive abilities of pathogens to antibiotics, the effect of genotypic changes on the immune response of the host, and the in vivo characterization of infection. Our current implementation of scDual-Seq does have the limitation of a high MOI which might have masked certain infection stages and phenotypes due to a potential variable number of bacteria in the infected macrophages. As previously described, constitutive GFP expression in the infected macrophages is not sufficiently precise to allow us to robustly conclude the number of bacteria infecting each macrophage; for example, starting with an MOI of 25, the GFP+ cells contain a range of 1–10 colony forming units (CFU) [3] Further work, perhaps using a microfluidics platforms [23, 24] to enable the analysis of a massive cohort of host–pathogen encounters, will address lower MOIs for a more accurate and comprehensive description of infection. Overall, the ability to capture both the pathogen and host transcription programs at the level of individual cells will be important for understanding the relationships among the different states of infection.

## Methods

### The scDual-Seq method

The SuperScript II Double Strand cDNA synthesis kit is used to convert the mRNA to double-stranded DNA with the following modifications: RT is performed in one-tenth volume (a total of 2 μL), as described in the protocol for random hexamer. After RT, RNase treatment is performed using a RNase cocktail enzyme mix (Life Technologies). A total of 0.5 μL mix containing 0.05 enzyme units is added and incubated at 37 °C for 30 min. 2X AMPure beads are added, incubated at room temperature for 20 min, and washed with 80% EtOH. The sample is then re-suspended in 1.5 μL 5X Tailing buffer and 5 mM dATP and incubated at 94 °C for 2 min. A total of 1 μL mix containing 2 units of terminal deoxynucleotide transferase (Life Technologies) is added to the sample, incubated at 37 °C for 30 min, and inactivated at 65 °C for 10 min. The second strand synthesis is performed in a final volume of 10 μL as described in the protocol with a modified CEL-Seq2 primer:

CGATTGAGGCCGGTAATACGACTCACTATAGGGGTTCAGAGTTCTACAGTCCGACGATCNNNNNAGACTCTTTTTTTTTTTTTTTTTTTTTTTTV

After second strand synthesis, the samples are pooled together and cleaned using 1.2X AMPure beads. IVT is performed in two-fifths reaction volume as described in the Ambion Message AMP II kit for 13 h.

### Measuring the sensitivity of scDual-Seq

We tested scDual-Seq on clean RNA of both mouse and *Salmonella* prepared as follows. Mouse RNA was isolated from CGR8 embryonic stem cell (ESC) line and prepared using a TRIzol extraction and treated with RQ1 RNase-free DNase (Promega) according to the manufacturers’ protocols. RNA cleanup was done with AMPure RNAClean beads. To extract *Salmonella* RNA, *Salmonella* were grown in LB and lysed by bead beating and RNA was extracted by the Qiagen RNeasy kit. RNA concentration was measured using Qubit, and diluted for technical replicates RNA in four different dilutions: 10 pg of mouse RNA mixed with 10 pg, 1 pg, 0.1 pg or 0.01 pg of *Salmonella* RNA, respectively, each in five replicates. All 20 samples were processed using scDual-Seq. As a side-by-side comparison, five replicates of the 10 pg of mouse RNA mixed with 0.01 pg of *Salmonella* were processed using the CEL-Seq2 protocol [12].

### Mice, cell lines, and bacterial strains

C57BL/6 WT mice were obtained from Jackson Laboratory. All animals were housed and maintained in a conventional pathogen-free facility at the Massachusetts General Hospital. All experiments were performed in accordance to the guidelines outlined by the MGH Committee on Animal Care. All *Salmonella typhimurium* strains used in this study were derived from the wild-type strain SL1344.

### Bone marrow-derived macrophage (BMDM) infection with *Salmonella*

Cultures of *S. typhimurium* labeled with GFP (pFPV25.1; Addgene) were grown in Luria-Bertani (LB) medium at 37 °C shaken at 250 rpm for 16 h (overnight culture). One milliliter from the overnight culture was washed in PBS and incubated for 1 h with pHrodo dye (Life Technologies) at room temperature in 100 mM sodium bicarbonate. *S. typhimurium* was then washed three times with HBSS and OD600 was measured. BMDMs were infected at an MOI of 50:1 and spun down for 5 min at 250 g. After 30 min, cells were washed with media containing 15 μg/mL gentamicin to remove *S. typhimurium* that were not internalized. We sorted 96 individual cells unexposed to *Salmonella*, individual cells 2.5, 4, and 8 h after infection. At each time-point cells were lifted from plates and sorted using FACS into 96-well plates containing 4.5 μL lysis buffer (TE containg 5%NP-40 and RNAse inhibitor) [3]. We repeated the experiment with unexposed cells and cells 4 h after infection; we sorted 40 individual exposed cells and 40 unexposed cells. Sorted cells were DNase treated with 1 uL enzyme mix of 0.2 U of DNase I at 65 °C for 5 min, cleaned with 1.8X RNAClean beads, and eluted with 1.2 μL of primer-dNTP mix before continuing to the RT step of scDual-Seq. Overnight culture *Salmonella*: 1 mL from the overnight culture was washed with PBS and RNA was extracted using phenol chloroform. A total of 1 ng of clean RNA was used as starting amount for the scDual-Seq protocol.

### Sequencing

Paired-end sequencing was performed on the HiSeq 4000 in high-throughput mode, 50 bases for read 1, seven bases for the Illumina index, and 50 bases for read 2. Read 2 was trimmed for 35 bases before mapping. On average, each single cell had 2.4 million reads. For the dilution experiment (Fig. 1), paired-end sequencing was performed on the HiSeq 2500 in rapid mode, 15 bases for read 1, seven bases for the Illumina index, and 36 bases for read 2.

### Data analysis and statistics

The initial analysis of the scDual-Seq sequenced reads was done using the CEL-Seq2 pipeline [12]. Reads were mapped to the mouse and *Salmonella* transcriptomes. In the last step, only reads mapping to the reverse strand are counted, since scDual-Seq produces stranded reads (htseq_wrapper; extra_params = −s reverse). In our first experiment, we filtered out cells with < 10,000 unique transcripts and with correlation of < 0.8 with at least ten other cells; we were left with 63 unexposed cells, 57 infected at 2.5 h, 59 infected at 4 h, and 42 infected at 8 h. In the second experiment, after filtering out single cells with < 20,000 unique transcripts, we were left with 33 unexposed cells and 38 infected cells. We used binomial statistics to convert the number of UMIs into transcript counts [13] and normalized to give read count in transcripts per 10,000.

### Gene Ontology analysis

Based on the tSNE plot shown in Fig. 2b, we defined two groups of cells: partially induced and induced cells. For each group, we identified 50 genes that are expressed highest in that group and with the lowest *P* value for differential expression between the two groups (Kolmogorov–Smirnov test). For each gene set we computed the enrichment for gene ontology terms (hypergeometric distribution) using annotations from Ensembl [25].

### Regulon expression analysis

For each cell, we summed the number of unique transcripts belonging to the same regulon [17] into a collective regulon expression and normalized to transcripts per thousand. For each defined group (partially induced, Class I, and Class II) we performed the Wilcoxon rank-sum test for each regulon comparing the expression level (tpm) between the group and the two other groups (for example, partially induced compared with Class I and Class II). Only regulons with higher expression levels in this group and a significant *P* value (*P* < 0.05) were selected for further analysis. Expression levels (tpm) for each of the selected regulons were averaged for each group. Finally, the mean values were normalized across the three groups, and ordered using ZAVIT [26, 27]. Correlation coefficients between transcriptomes (Fig. 3b) were computed based upon their *Salmonella* regulon expression levels (log_10_ tpm) of all infected cells. A Wilcoxon rank-sum test was used to compare the correlations between the partially induced and Class I cells with respect to the partially induced and Class II cells.

### Pseudo-time analysis

We used the ZAVIT method [26, 27] to order the cells based on tSNE-1 and tSNE-2 values generated in Fig. 2e. The values were coerced to a smooth path by a moving mean over 50 radius values. The length of the path going through the cells in tSNE-1, tSNE-2 space was calculated and used as pseudo-time. For SPI-1 and SPI-2 expression profiles in single cells, we filtered out cells with no expression for both SPI-1 and/or SPI-2 regulons. The expression profiles were ordered by the pseudo-time, normalized, and smoothed.

## Additional files

Additional file 1: scDual-Seq protocol. This file includes the detailed scDual-Seq protocol. (PDF 265 kb)

Additional file 2: Supplementary figures. This file includes four supplementary figures. (PDF 3997 kb)
