# Supplementary material for: scDual-Seq: mapping the gene regulatory program of Salmonella infection by host and pathogen single-cell RNA-sequencing
Source: Genome Biol. 2017 Oct 27;18:200. doi: 10.1186/s13059-017-1340-x (PMC5658913; doi:10.1186/s13059-017-1340-x)
Supplement: Supplementary file 1 — scDual-Seq protocol. This file includes the detailed scDual-Seq protocol. (PDF 265 kb) [file 13059_2017_1340_MOESM1_ESM.pdf]

scDual-Seq protocol

1. Sorting and breaking cell open:

Prepare lysis buffer:

TE	945 µl
NP40	50 µl
RNase inhibitor	5 µl

1. Add 5 uL lysis buffer to each well in a LoBind 96 well plate.
2. Sort single cells into LoBind 96 plate.
3. Freeze in -80°C.

Prepare DNAse mix:

DNAse Buffer	0.6µl
DNAse I	0.2µl
DDW	0.2µl
	1 ul

- Add 1ul of DNAse mix to each well in a LoBind 96 well plate
- Incubate the plate in a thermal cycler as follows:
  - 37C 5 min
  - 75C 3.5 min
  - move to ice (>1 min.).

2. RNA cleanup and primer annealing:

Prepare primer mix (Multiple depending on the number of samples you have):

Random hexamers (6µM)	1µl
dNTPs (10mM)	1µl
DDW	8µl
	12µl

- Prewarm XP RNA beads to room temperature.
- Vortex AMPure XP Beads until well dispersed
- Add 1.8X RNA XP beads (10.8 µl), Mix entire volume up ten times to mix thoroughly.
- Incubate at R/T for 10 min.
- Place on magnetic stand for at least 5 min, until liquid appears clear.
- Remove and discard the supernatant.
- Add 80µl freshly prepared 70% EtOH.
- Magnet switch to wash. Remove and discard supernatant.
- Repeat the wash to a total of 2 times.
- Air dry the beads for 10 min
- Resuspend in 1.2µl primer mix.
- Incubate 5 min. at 65C (with lid of thermal cycler set to 70C) → ice (>1 min.).

- Spin at maximal speed for a few seconds to collect as many droplets as possible before next step, and then return to ice.
- Preheat thermocycler to 25C.

3. Reverse Transcription (SSII):

In a new tube, mix the following RT mix (prepare on ice! Multiply each reagent by the number of samples):

1st strand buffer	0.4µl
DTT	0.2ul
RNaseOUT	0.1µl
SSII enzyme	0.1µl
	0.8µl

- Pipette 6-8 times, spin.
- Add 0.8 µl RT mix each reaction.
- Pipette 6-8 times, spin. (Total volume- 2µl)
- 25C 10 min. → ice (>1 min.).
- Pre-heat the thermal cycler to 42C (lid at 50C)
- 42C for 60 min. → ice (>1 min.).
- 70C for 10min. → ice (>1 min.)

4. RNase mix:

- preheat thermocycler to 37C.
- Mix 0.1µl RNase mix with 0.4µl DDW (Multiply by the number of samples)
- Add 0.5ul diluted RNase mix to the RT product
- 37C for 30 min. → ice (>1min.)

5. cDNA cleanup:

Prepare TdT mix (Multiply by the number of samples):

DDW	0.75 µl
5X tailing buffer	0.5µl
dATP 5mM	0. 25µl
	1.5µl

- Prewarm XP beads to room temperature.
- Vortex AMPure XP Beads until well dispersed
- Mix 2.5 µl of DDW with 2X (10 µl) of XP beads per sample.
- Mix beads with the sample ten times to mix thoroughly.
- Incubate at R/T for 20min.
- Place on magnetic stand until liquid appears clear.
- Remove and discard the supernatant.
- Add 80µl freshly prepared 80% EtOH.
- Remove and discard supernatant.
- Repeat the wash to a total of 2 times.
- Air dry the beads for 10 min
- Resuspend in 1.5µl TdT mix

- Pipette 6-8 times.
- 94C for 2 min (lid 100C) → ice
- Quick spin
- Mix 0.1µl TdT with 0.9µl DDW (Multiply by the number of samples).
- Add 1µl of diluted TdT to each sample. (total volume 2.5µl)
- 37C → 30 min
- 65C → 10 min → ice

6. 2nd strand synthesis (SuperScript II):

5X 2nd strand buffer, dNTP mix, CEL-Seq2 primers → Thaw on ice, spin 5 sec, place on ice.
Pre-heat the thermal cycler to 70C with lid at 100C.

- Add 0.5 µl CEL-Seq primers (2.5ng/ul).
- Pipette 6-8 times, spin.
- 70C at 10 min → ice
- Pre-heat the thermal cycler to 16C with no heated lid.

Prepare 2nd strand mix (on ice, Multiply by the number of samples):

DDW	4.2 µl
5X 2st strand buffer	2µl
dNTP Mix	0.3µl
DNA polymerase	0.4µl
Ligase	0.1µl
	7.0µl

- Pipette 6-8 times, spin.
- Add 7.0 µl 2nd strand mix to the RT tube.
- Pipette 6-8 times, spin. (Total volume- 10µl)
- 16C for 2 hours (no heated lid, make sure lid is not hot) → ice.

7. 1.2X cDNA cleanup with beads:

- Prewarm beads and beads buffer to room temperature.
- Vortex AMPure XP Beads until well dispersed
- Pool all cells that are to go to same IVT. Should have ~10µl from each cell.
- Place on magnetic beads for at least 5 min, until liquid appears clear.
- Transfer the supernatant to a new tube.
- Split the supernatant to ~200ul per tube
- Add 0.2X volume of beads (~40ul) and 1X volume of beads buffer(~100ul) to your cDNA. Incubate at room temperature for 10 min.
- Place on magnetic stand for at least 5 min, until liquid appears clear.
- Remove and discard the supernatant.
- Add 200µl freshly prepared 80% EtOH.
- Incubate at least 30 seconds, then remove and discard supernatant without disturbing beads.
- Add 200µl freshly prepared 80% EtOH

- Incubate at least 30 seconds, then remove and discard supernatant without disturbing beads.
- Air dry beads for 15 min, or until completely dry.
- Resuspend each tube with 6.4µl DDW. Pipette entire volume up and down ten times to mix thoroughly.
- Incubate at room temperature for 2 min.
- Pool together tubes that go to the same IVT.
- Keep the beads in for the next step!

8. IVT (Ambion):

T7 10X buffer, T7 ATP, T7 CTP, T7 GTP, T7 UTP, → Thaw on ice, spin 5 sec, place on ice. Thermal cycler programed to 37C for 13 hr and 4C hold.

IVT mix (on ice):

T7 ATP	1.6µl
T7 CTP	1.6µl
T7 GTP	1.6µl
T7 UTP	1.6µl
T7 10X buffer	1.6µl
T7 Enzyme mix	1.6µl
	9.6µl

- Pipette 6-8 times, gently vortex, spin.
- Add 9.6µl IVT mix to the sample tube.
- Pipette 6-8 times, spin. (Total volume- 16µl)
- Place in the thermal cycler- 37C for 13 hr (lid at 70) and 4C hold.

9. exosap and Fragmentation

- Pre-heat the thermal cycler to 37C with lid at 50C.
- Add 6ul of exosap to the 16µl of aRNA
- 37C for 15 min → ice
- Add 5X fragmentation buffer (5.5ul) to 22 ul of aRNA.
- Incubate at 94C for 3 min (lid at 105).
- Place on ice immediately.
- Add 2.5ul stop buffer

10. 1.5X RNA beads cleanup:

- Prewarm XP RNA beads to room temperature.
- Vortex AMPure XP Beads until well dispersed
- Place the fragmented aRNA tube on the magnetic beads for at least 5 min, until liquid appears clear.
- Transfer the supernatant to a new tube.
- Add 1.5X RNA XP beads, mix entire volume up ten times to mix thoroughly.
- Incubate at R/T for 10min.
- Place on magnetic stand for at least 5 min, until liquid appears clear.

- Remove and discard the supernatant.
- Add 200µl freshly prepared 70% EtOH.
- Incubate 30 sec, remove and discard supernatant.
- Repeat the wash to a total of 2 times.
- Air dry the beads for 10 min
- Resuspend in 5.5µl nuclease free water and incubate at RT for 2 minutes.
- Place on magnetic stand for 1 min, until liquid appears clear.
- Transfer 5 uL supernatant to a new tube.

<u>Check aRNA amount and quality:</u>

Optional – If it's the first time you do the protocol, or you are using different starting material than usual, it is recommended to check the aRNA on a pico RNA bio-analyzer chip (see more info in CEL-Seq2 protocol).

<u>11. Reverse Transcription (RTP6N):</u>

- 10mM dNTPs, RTP6N, 5X 1st strand buffer, 100 mM DTT → Thaw on ice, spin 5 sec, place on ice.
- Pre-heat the thermal cycler to 65C (with lid 100)
- In a new tube, mix the following:

Treated aRNA	5µl
RTP-6N	1µl
dNTPs	0.5µl
	6.5µl

- Pipette 6-8 times, spin.
- 65C for 5 min (with lid 100) → ice.
- Pre-heat the thermal cycler to 25C
- RT mix (prepare on ice! Multiply each reagent by the number of samples):

5X 1st strand buffer	2µl
100mM DTT	1µl
RNase inhibitor	0.5µl
SuperScriptII Reverse transcriptase	0.5µl
	4µl

- Pipette 6-8 times, spin.
- Add 4µl RT mix to the aRNA treated tube.
- Pipette 6-8 times, spin. (Total volume- 10.5µl)
- 25C for 10min → ice
- Heat thermal cycler to 42C
- 42C for 50min (lid 50C) → ice.

<u>12. PCR amplification:</u>

RP1, RPI, PCR mix→ Thaw on ice, spin 5 sec, place on ice.
Use only half of the RT reaction (5ul) the other half can be kept in -20.

To half a reverse transcription reaction add 20µl of the following mix:

Ultra-pure Water	5.5µl
PCR mix	12.5µl
RNA PCR Primer (RP1, from Illumina kit)	1µl
indexed RNA PCR Primer (RPIX, from Illumina kit)	1µl
	20µl

- Pipette 6-8 times, spin, ice.

Set the thermal cycler as follows:
30 sec at 98C
12 cycles of: 10 sec 98C
30 sec at 60C
30 sec at 72C
10 min at 72 C
Hold at 4C

13. Bead Cleanup (double 1X) of PCR products:

- Prewarm beads to room temperature.
- Vortex AMPure XP Beads until well dispersed.
- Add 25µl to each 25µl PCR reaction. Mix entire volume up ten times to mix thoroughly.
- Incubate at room temperature for 15 min.
- Place on magnetic stand for at least 5 min, until liquid appears clear.
- Remove and discard 45µl of the supernatant.
- Add 200µl freshly prepared 80% EtOH.
- Incubate at least 30 seconds, then remove and discard supernatant without disturbing beads.
- Add 200µl freshly prepared 80% EtOH
- Incubate at least 30 seconds, then remove and discard supernatant without disturbing beads.
- Air dry beads for 15 min, or until completely dry.
- Resuspend with 25µl DDW. Pipette entire volume up and down ten times to mix thoroughly.
- Incubate at room temperature for 2 min.
- Place on magnetic stand for 5 min, until liquid appears clear.
- Transfer 25µl of supernatant to new tube.

- Repeat as above but eluting in 12µl nuclease free water at the end, transferring 10µl to a new tube.

14. Check library amount and quality:

- Check concentration of DNA by Qubit, 1µl should be enough to measure using the high sensitivity reagent; expected concentration is at least ~1ng/µl.
- Run 1µl of each sample on Bioanalyzer using a high sensitivity DNA chip to see size distribution. Expected peak at 200-400bp (See Bioanalyzer plot for example).

Reagents:
TE- 10mM Tris pH8, 1mM EDTA
NP40- Fisher PI28324
RNaseOUT – Invitrogen 10777-019
DNase- RQ1 Promega PR-M6101
Ultra-pure RNase free water
Random hexamers 6uM
dNTPs mix 10mM
RNAClean XP beads - Beckman coulter A63987
Ethanol
SuperScriptII- Invitrogen 18064-014
RNase mix- Ambion AM2286
5X tailing buffer- 50 mM Tris-HCl (pH 8.4), 125 mM KCl, 7.5 mM MgCl2
5X FS buffer SuperScriptII, 125mM KCL, 11.25mM MgCL2
dATP 5mM
AMPure XP beads – Beckman Coulter A63880
Beads buffer - 20% PEG8000, 2.5M Nacl
TdT- fisher 10533065
Second strand buffer – Invitrogen 10812-014
DNA Plymerase I (E. coli) – Invitrogen 18010-025
E. coli DNA ligase – Invitrogen 18052-019
IVT kit- MEGAscript T7 Transcription Kit – Ambion AM1334
ExoSAP-IT For PCR Product Clean-Up – Affymetrix 78200
Fragmentation buffer: 200mM Tris-acetate, pH 8.1, 500 mM KOAc, 150 mM MgOAc
Fragmentation stop buffer: 0.5 M EDTA pH8
RT random primer (same as in CEL-Seq2)- GCCTTGGCACCCGAGAATTCCANNNNNN
PCR mix- Phusion® High-Fidelity PCR Master Mix with HF Buffer – NEB M0531
RNA PCR primers (sequences available from Illumina TruSeq small RNA kit)
