# Supplementary material for: scDual-Seq: mapping the gene regulatory program of Salmonella infection by host and pathogen single-cell RNA-sequencing
Source: Genome Biol. 2017 Oct 27;18:200. doi: 10.1186/s13059-017-1340-x (PMC5658913; doi:10.1186/s13059-017-1340-x)
Supplement: Supplementary file 2 — Supplementary figures. This file includes four supplementary figures. (PDF 3997 kb) [file 13059_2017_1340_MOESM2_ESM.pdf]

Supplementary Figure 1

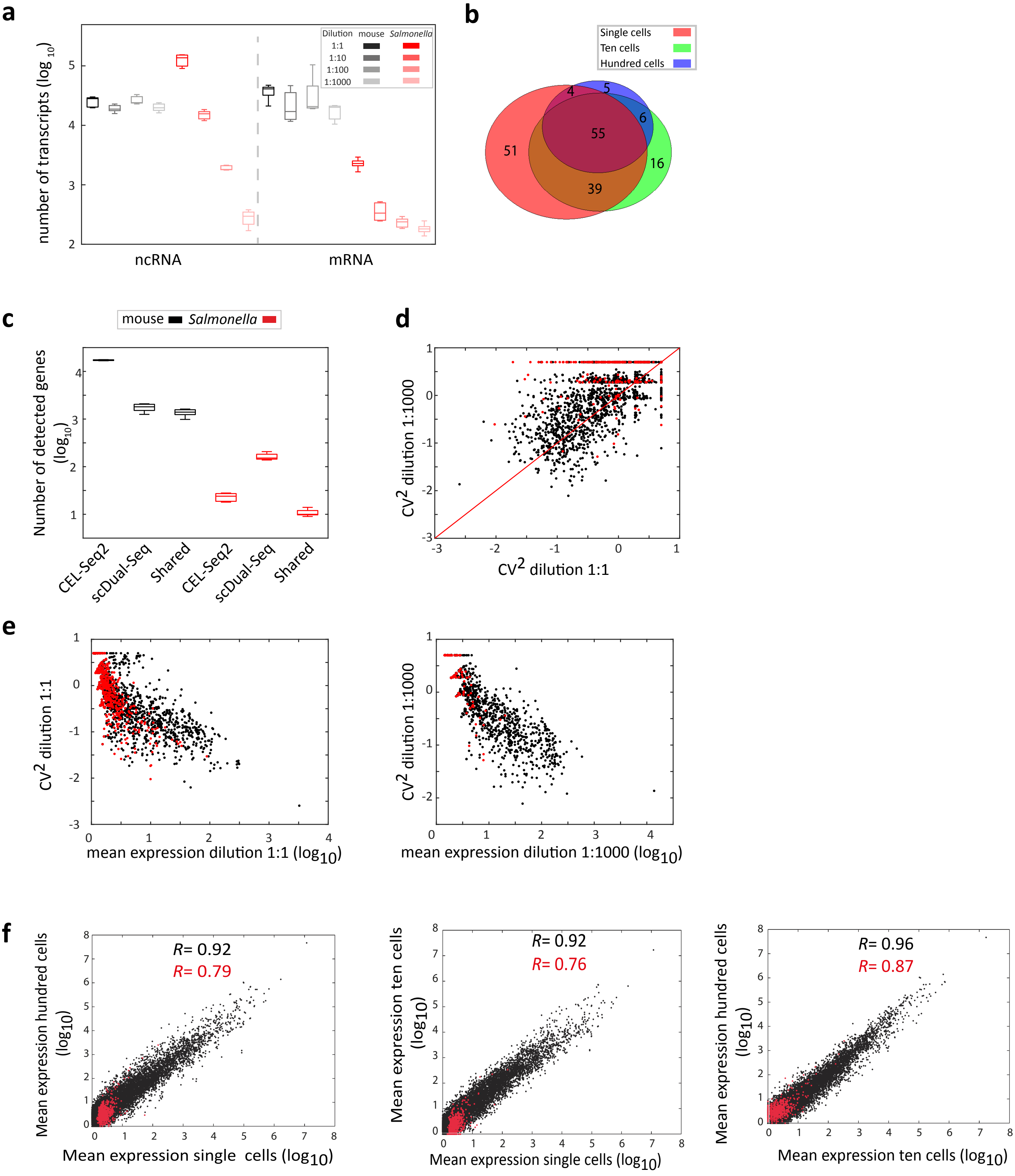

Supplementary Figure 2

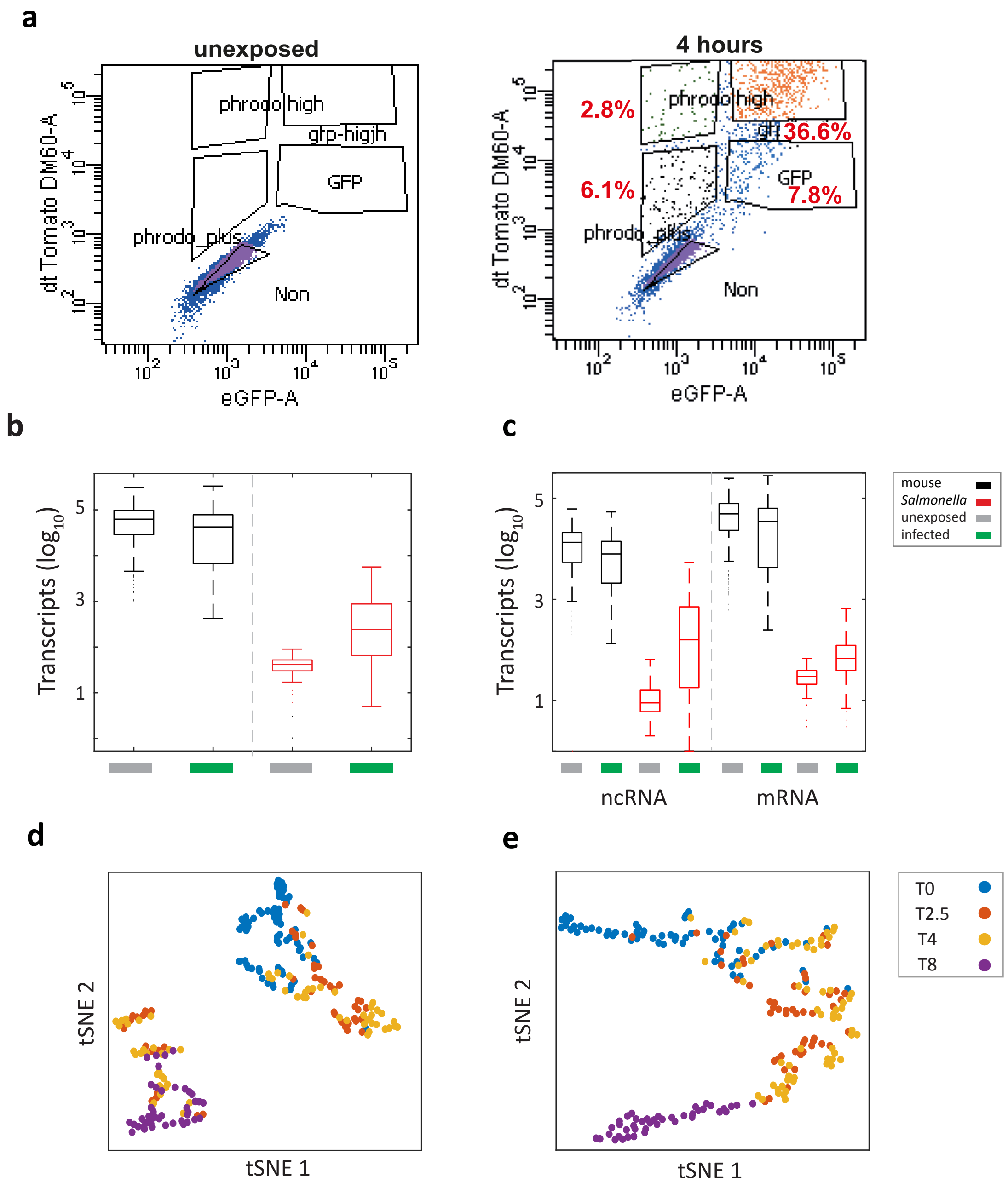

Supplementary Figure 3

a

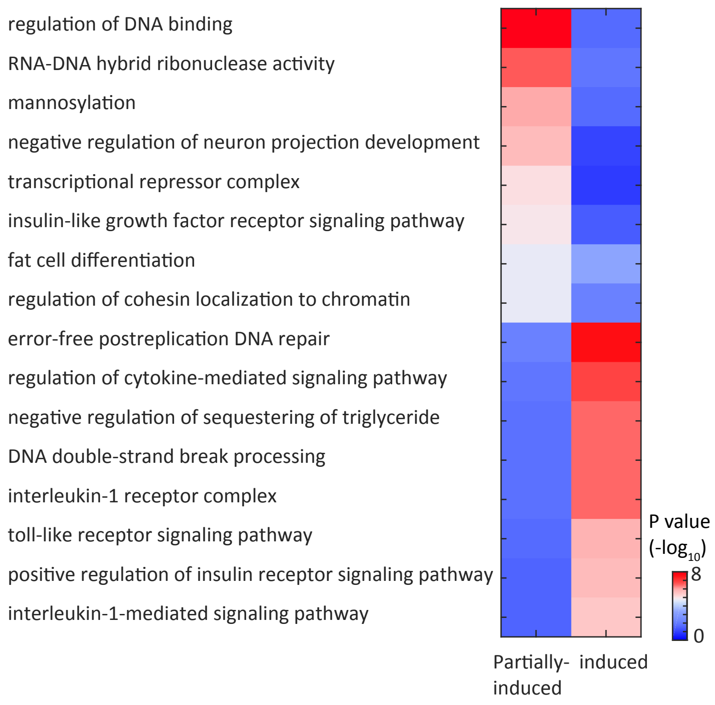

b

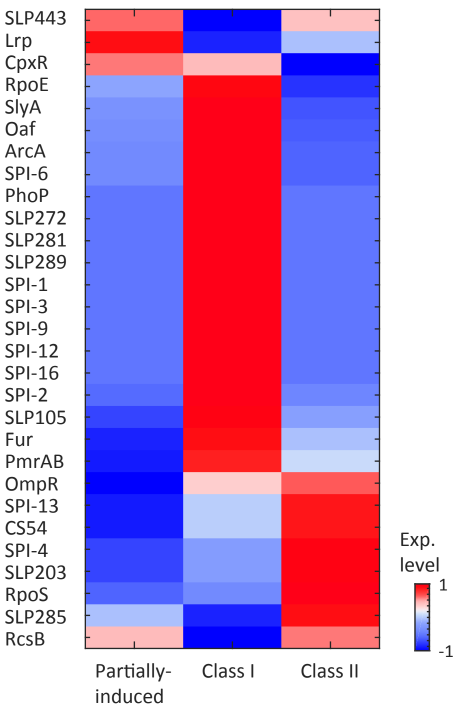

Supplementary Figure 4

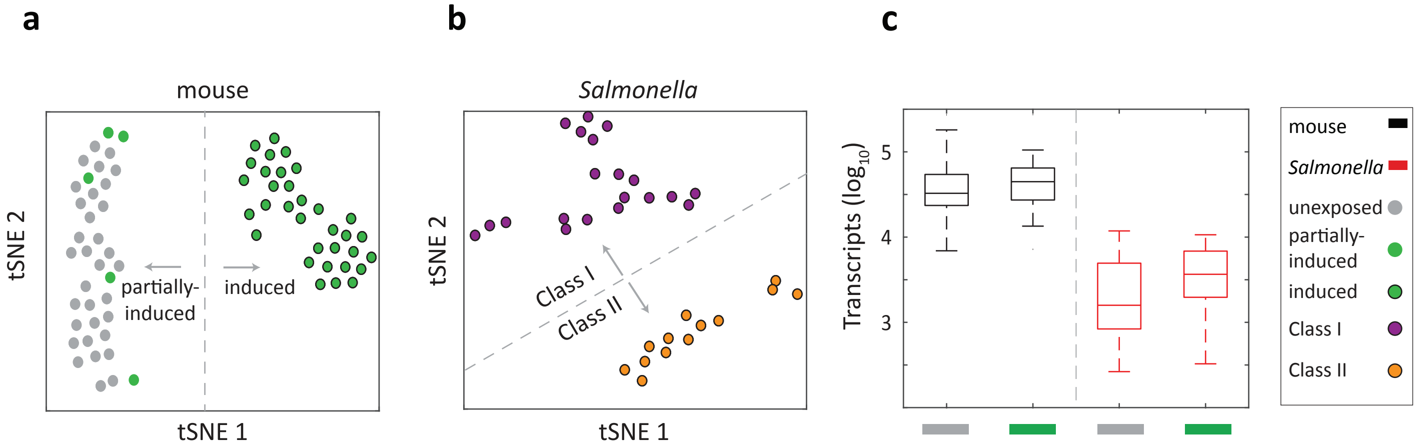

SUPPLEMENTARY FIGURE CAPTIONS

Figure S1: The scDual-Seq method. a Boxplots indicating the number of unique non-coding RNA and mRNA transcripts for different mouse and *Salmonella* dilutions, as described in Figure 1. **b** Venn diagram showing the overlap among the sets of *Salmonella* genes that are differentially expressed between the overnight culture of *Salmonella* and the single, 10, or 100 infected cells. **c** Boxplots indicating the number of detected genes (expression higher than 0) using CEL-Seq2, scDual-Seq and the number of shared detected genes between the two methods for mouse *Salmonella* 1:1000 dilution. **d** A comparison between the squared coefficients of variation for technical replicates, mouse to *Salmonella* ratio, as described in Figure 1. **e** A comparison between the squared coefficients of variation for technical replicates compared to the mean gene expression of the 1:1 and 1:1000 mouse:*Salmonella* sample. **f** Correlation between the mean expression of mouse genes (black) and *Salmonella* genes (red) between single, 10, and 100 cells. We found good correspondence of *Salmonella* transcriptomes between the single-cell data and population-level data, as well as between the 10 cell and 100 cell population data, demonstrating the accuracy of the single-cell measurements of bacterial transcripts.

Figure S2: Querying scDual-Seq in infected cells. a FACS plots of fluorescently labeled BMDMs. Unexposed cells (left) have no detected fluorescence, while exposed cells four hours after infection (right) harboring live bacteria are labeled with both Phrodo and GFP (upper right gate). The percentages indicate the frequency of each population. **b** Boxplots of number of mouse (black) and *Salmonella* (red) transcripts in individual cells unexposed (grey) or exposed to *Salmonella* (green). **c** Boxplots indicating the number of unique non-coding RNA and mRNA transcripts for individual cells unexposed (grey) or exposed to *Salmonella* (green). **d** tSNE plot

of single cells positioned as Fig. 2b. The color indicates the time-point of cells collection- unexposed (T0 blue), 2.5h after infection (T2.5 red), 4 after infection (T4 yellow), 8h after infection (T8 purple). **e** tSNE plot of individual cells positioned as Fig 2e. The color indicates the time-point of cells collection as in **d**. The different classes were not distinguished by cell cycle state (analysis not shown).

Figure S3: Functional annotation of the identified subpopulation in mouse and *Salmonella*.

a Gene Ontology (GO) terms of differentially expressed mouse genes ($P<0.00005$) between induced and non-induced (see Methods). **b** Expression level of differentially expressed regulons ($P<0.05$) between each subpopulation and the other two (See Methods). Class I *Salmonella* transcriptome has high expression of virulent genes of several SPI regulons, such as SPI-2, as well as other regulatory genes such as PhoP. SPI-1 and SPI-4 have opposite expression patterns while a previous work studying their regulation in culture and infecting epithelial cells showed that they are co-expressed[29]. SPI-4 is required for adhesion and adhesive *Salmonella* may have been eliminated by our antibiotics treatment.

Figure S4: Assaying the reproducibility and sensitivity of scDual-Seq.

a tSNE plot of individual cells computed based on 665 mouse genes with high variation (mean/median >1.5). Color indicates unexposed (grey) infected time-point 4h (green) and induced (black circle). **b** tSNE plot of induced single cells computed based on 32 *Salmonella* regulons. The color indicates Class I (purple) Class II (orange). **c** Boxplots of number of mouse (black) and *Salmonella* (red) transcripts in individual cells unexposed (grey) or exposed to *Salmonella* (green) in the second experiment. Analyzing the unexposed single cells in the second

experiment, we found an average of 225 transcripts that mapped to *Salmonella* genes (Figure S2c). We suspected that since those cells were sorted to the same plate with the exposed cells, environmental contamination such as aerosols in the sorting process, can account for these transcripts as shown[30] for single-cell analysis of bacterial nucleic acids.
